# AAV9 gene therapy optimization for SMARD1/CMT2S: safety and long-term efficacy comparison of two vectors in a SMARD1 preclinical model

**DOI:** 10.1186/s12929-025-01204-z

**Published:** 2026-01-04

**Authors:** Elisa Pagliari, Alessia Anastasia, Floriana Bellandi, Manuela Garbellini, Jessica Ongaro, Michela Taiana, Giacomo P. Comi, Linda Ottoboni, Julieth Andrea Sierra-Delgado, Shibi Likhite, Kathrin C. Meyer, Monica Nizzardo, Stefania P. Corti

**Affiliations:** 1 Department of Pathophysiology and Transplantation, University of Milan, Milan, Italy; 2 Neurology Unit, IRCCS Foundation Ca’ Granda Ospedale Maggiore Policlinico, Milan, Italy; 3 Center for Gene Therapy, Abigail Wexner Research Institute, Nationwide Children’s Hospital, Columbus, OH USA; 4 Department of Physical Medicine and Rehabilitation, NextGen Precision Healthm, University of Missouri School of Medicine, Columbia, MO 65211 USA; 5 Alcyone Therapeutics, Lowell, MA USA; 6 Foundation IRCCS Ca’ Granda Ospedale Maggiore Policlinico, Neuromuscular and Rare Diseases Unit, Milan, Italy

**Keywords:** Motor neuron diseases, Adeno-associated virus, Gene therapy, Viral vectors

## Abstract

**Background:**

Mutations in the *Immunoglobulin Mu DNA Binding Protein 2* (*IGHMBP2*) gene cause Spinal Muscular Atrophy with Respiratory Distress type 1 (SMARD1), a rare, infantile, and fatal motor neuron disease, as well as the milder Charcot-Marie-Tooth disease type 2S (CMT2S). Gene therapy has emerged as a promising approach to correcting IGHMBP2 loss in SMARD1 models, but critical challenges remain.

**Methods:**

In this study, we compared the efficacy of two novel, optimized adeno-associated virus 9 (AAV9)-*IGHMBP2* vectors, utilizing either the Chicken β-Actin (CBA) or a truncated form of the methyl-CpG-binding protein 2 (MeCP2) promoter (P546), in the SMARD1 murine model via intracerebroventricular delivery. Treated mice survival, histopathological and molecular profile were analyzed.

**Results:**

Corroborating previous findings, both constructs effectively rescued the pathological phenotype, significantly improving survival, body weight, and motor function while preserving motor neurons and neuromuscular junctions. Notably, histopathological and RNA sequencing analyses revealed, for the first time, inflammatory marker alterations in the SMARD1 spinal cord, which resolved following treatment. A comparative analysis of the two vectors demonstrated superior long-term efficacy of the P546-promoter construct.

**Conclusion:**

ICV gene therapy approach can effectively rescue SMARD1 pathological hallmarks, including astrogliosis and microgliosis. Moreover, P546-promoter construct is superior in terms of safety profile and long-term therapeutic efficacy.

**Graphical abstract:**

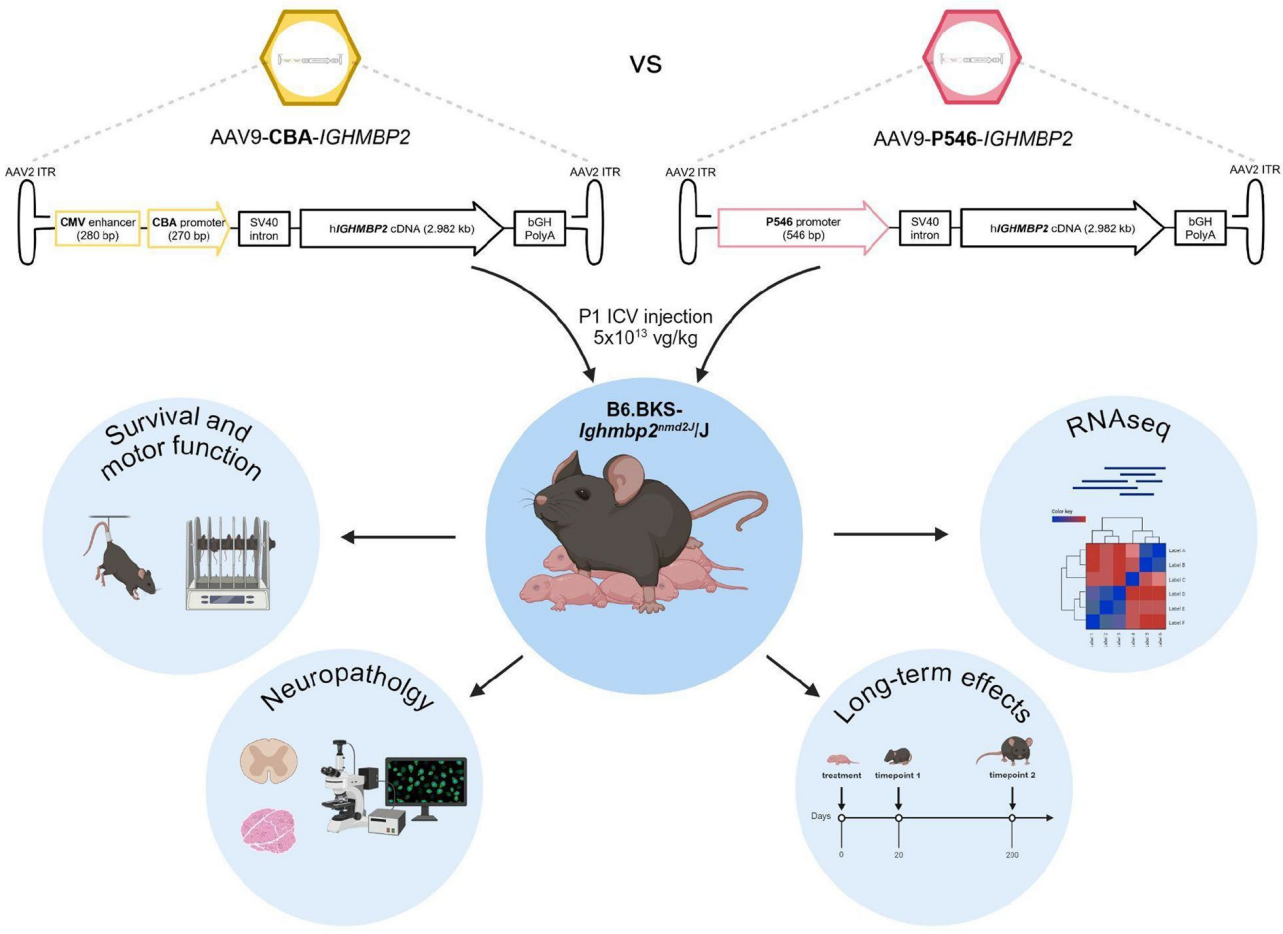

**Supplementary Information:**

The online version contains supplementary material available at 10.1186/s12929-025-01204-z.

## Introduction

Spinal Muscular Atrophy with Respiratory Distress type 1 (SMARD1, OMIM #604,320) is a rare genetic disease with autosomal recessive inheritance [1]. The prevalence is estimated to be less than 1:1,000,000 births [2]. SMARD1 resulted from mutations in the *Immunoglobulin Mu DNA Binding Protein 2* (*IGHMBP2*) gene which encodes for the ubiquitous IGHMBP2 protein, classified as an ATP-dependent helicase with low tissue specificity, and a multifunctional, even not still completely understood, role (transcription, translation, replication, rRNA biogenesis, and tRNA metabolism) [3–5]. Mutations do not exhibit a clear genotype–phenotype correlation, and their type is heterogeneous, limiting the application of direct specific mutation correction approaches [6]. Moreover, it is well recognized that *IGHMBP2* variants can also result in the milder axonal neuropathy Charcot-Marie-Tooth disease type 2S (CMT2S) [4]. Clinically, SMARD1 is a fatal motor neuron disease (MND) with infantile-onset, characterized by the selective degeneration of the spinal cord alpha motor neurons (α-MNs), resulting in hindlimb distal muscle weakness, and the distinct involvement of the diaphragm, which leads to early respiratory distress within the first six months of life and the necessity of ventilatory support, different from the classical Spinal Muscular Atrophy (SMA) form [7–9]. Life expectancy is significantly reduced to an average of 13 months, although there is considerable variability [10]. To date, no therapies have been approved and only supportive treatments are currently utilized.

The current therapeutic strategies under investigation aim to directly replace the mutated gene, modulate cryptic splicing by antisense oligonucleotide site, or to find complementary approaches *IGHMBP2*-independent [11–16]. Up to now, the most promising approach is gene therapy, which relies on the use of non-integrating vectors to deliver a healthy copy of the mutated gene, and that has already been approved for other similar pathologies as SMA (e.g., Zolgensma^®^) [17, 18]. In this regard, adeno-associated virus serotype 9 (AAV9) is considered a prime candidate vector for nervous system diseases, it is highly efficient in large animal species, and the biodistribution data from human patients treated with Zolgensma^®^ confirmed widespread transduction of the nervous system including motor neurons [19, 20]. We previously demonstrated the efficacy of AAV9-mediated gene therapy, administered by systemic intravenous (IV) injection, in rescuing the SMARD1 murine model phenotype when treated at postnatal day 1 [21], opening the path to other studies on gene therapy approach in SMARD1 models [22–24].

However, the gene therapy strategy requires further studies to be optimized—for example, in terms of dose, route of administration, therapeutic window, and viral vector design. In this study, we focus on the latter aspect, particularly the choice of promoter, a pivotal element in controlling transgene expression that could enable refined dosage and expression regulation. We selected an intracerebroventricular (ICV) injection, to deliver the vector directly into the spinal fluid, a more suitable route of injection for clinical trials to reduce the risk of high dose AAV toxicity [23, 26, 27].

Here we describe simultaneous blinded testing of two AAV9 gene therapy candidates that supported lead candidate selection for further IND-enabling studies, ultimately leading to the initiation of the ongoing first in human clinical trial for treatment of SMARD1/CMT2S [25] (NCT05152823).

The AAV9 vector candidates differed only in the promoter chosen to control expression of the *IGHMBP2* transgene. Of note, both promoters in question have already been used in other clinical applications before: Chicken β-Actin promoter (CBA) [20, 28] and the truncated form of the methyl-CpG-binding protein 2 (MeCP2) promoter (P546) [26, 27, 29]. Both promoters lead to ubiquitous expression in neurons and glial cells, but the CBA promoter leads to a strong expression profile particularly in astrocytes, while the P546 promoter allows a more moderate and physiological transgene expression levels across multiple cell types such as astrocytes and neurons.

While both promoters successfully ameliorated disease phenotypes, leading to the rescue of both motor neurons and muscle-related pathology, the P546 promoter had a more pronounced effect, especially at later timepoints post treatment. Moreover, we demonstrate, for the first time, inflammatory marker alterations in the SMARD1 spinal cord as disease aspect, which were also restored following treatment.

## Results

### Therapeutic efficacy of two different AAV9:*IGHMBP2* constructs in a SMARD1 mouse model

To refine the gene therapy strategy for SMARD1, we compared two AAV9 gene therapy vectors expressing the coding sequence of *IGHMBP2* under two different promoters which have already an established track record in clinical applications: the Chicken β-Actin promoter (CBA), and the P546 promoter (a truncated version of the MeCP2 promoter). Initial assessment of the therapeutic potential of these vectors involved treating SMARD1-affected pups (*nmd*) at postnatal day 1 (P1) via ICV injection with 5 × 10^10^ vector genomes/animal of either CBA, P546 or an empty vector (null) that we used as the negative control (Figure S1A-C). Blinding was performed by the team at Nationwide Children’s Hospital and maintained during the entirety of the study to ensure avoidance of bias. We confirmed the increased IGHMBP2 expression in the spinal cords at P20 obtained by both the two therapeutic vectors (Figure S2A). A key difference between the two constructs was clearly evident: the CBA promoter lead to stronger expression than the P546 promoter at this early time point, with protein amount significantly higher than the levels observed in wild-type (WT) mice (Figure S2B, CBA vs null *P* = 0.0036; CBA vs WT *P* = 0.0106); in contrast, while protein amount observed in the P546*-*treated group was higher than in the null-treated cohort, it reached a value comparable to the endogenous IGHMBP2 expression in WT animals (Figure S2B).

**Fig. 1:**
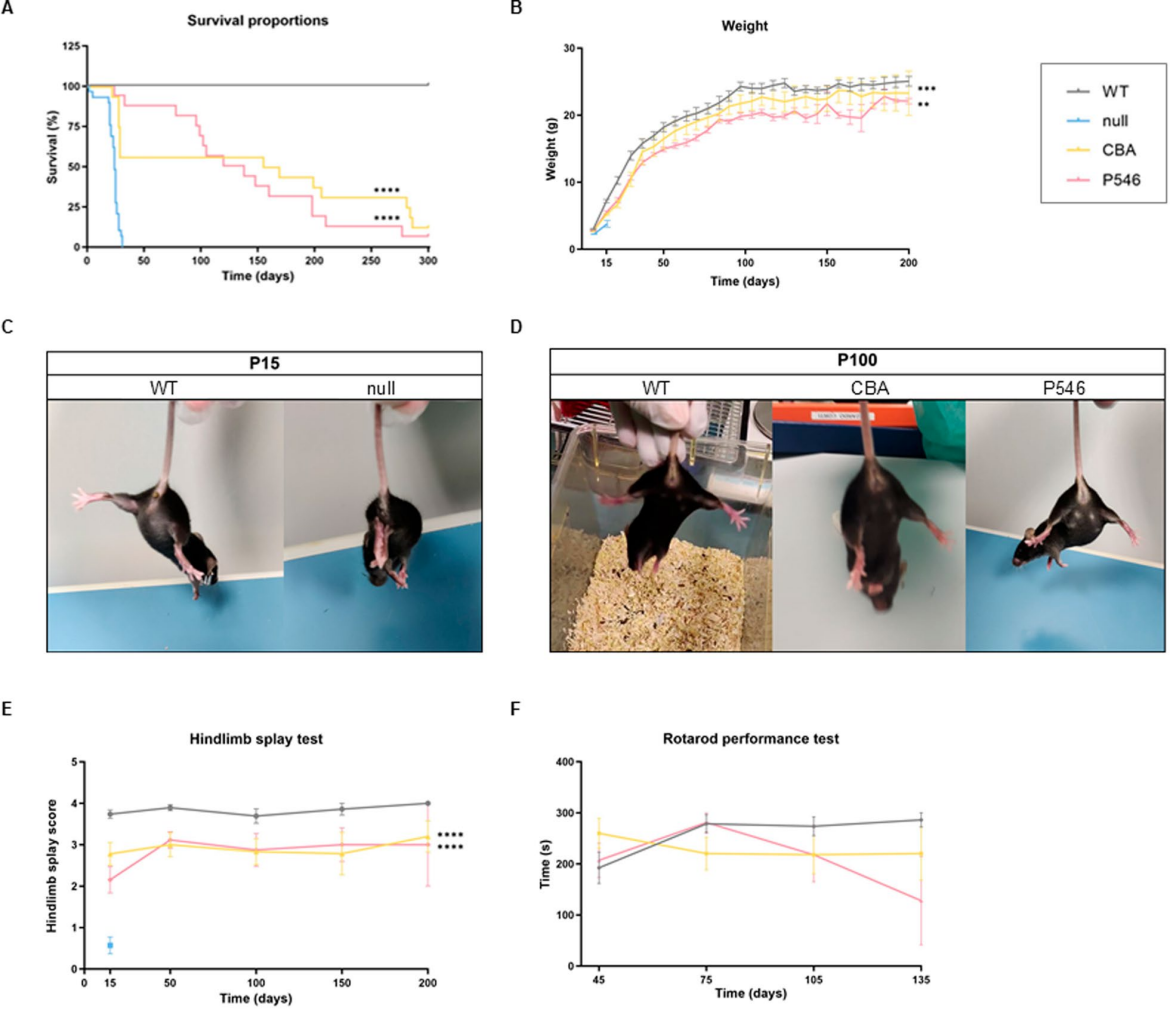
Survival and phenotypical test of *nmd* mice receiving gene therapy. Survival estimation and motor tests performed on WT (n = 28), and *nmd* mice treated with CBA (n = 28), P546 (n = 28), or null (n = 29) vectors. **A** Kaplan–Meier survival curve. **B** Body weight curve represented as mean grams value ± SEM. **C**–**D** Representative images of macroscopic phenotypical difference between WT and *nmd* null-treated mice at P15 (**C**) and among WT, CBA and P546 treated mice at P100 (**D**). **E** Hindlimb splay test score evaluated at different time points. The values are represented as mean value ± SEM. **D** Rotarod performance recorded monthly starting from P45. Statistical analysis were performed with Log-rank Mantel-Cox test (**A**) and one-way ANOVA followed by Dunnett’s multiple comparison test versus the null group (**B**–**E**). ****P < 0.0001, ***P < 0.001, **P < 0.01

We next examined the therapeutic impact on SMARD1 pathology by monitoring the survival and motor function of WT and *nmd* mice treated with CBA, P546 or null vectors. Survival analysis indicated a tremendous increase in lifespan for both the groups treated with *IGHMBP2* vectors (CBA and P546 vs null *P* < 0.0001), with median survivals of 162 days and 129 days respectively, compared to 24 days for null-treated mice (Fig. 1A), lifespan values equivalent to untreated *nmd* mice (not shown).

At gross examination, both therapeutic vectors resulted in body weight amelioration, with values comparable to WT littermates. In contrast, null-treated *nmd* mice exhibited early mortality, precluding multiple weight measurements (Fig. 1B, CBA and P546 vs null respectively *P* = 0.0005 and *P* = 0.0018).

A classical feature of *nmd* mice is the presence of muscle wasting and severe limb contracture already present in the early symptomatic phase of the disease [30] (Fig. 1C). Both the *IGHMBP2* treated *nmd* mice groups are almost unrecognizable from WT littermates at P100 (Video S1) showing significant improvement in the hindlimb muscles appearance (Fig. 1D) and in the hindlimb splay test during all their lifespan (Fig. 1E, CBA and P546 vs null both *P* < 0.0001), confirming the ability of both constructs into rescue motor functions [30]. Additionally, motor abilities and balance, assessed by the rotarod test, revealed that a significant proportion of mice treated with CBA or P546 could successfully perform the test, unlike their null counterparts which did not even reach the correct age to perform it (Fig. 1F).

Serological analysis revealed that gene therapy with both constructs did not negatively affect hepatic and kidney functionality (Figure S3), confirming the absence of toxicity signs, fundamental for the translational point of view. Notably, treatment resulted in normalization of glucose and alkaline phosphatase (ALP) levels that were deregulated in null mice.

### Neuropathological improvement in SMARD1 mice following gene therapy

To determine the effectiveness of the AAV9-based gene therapy in mitigating the typical neuropathological features of SMARD1, we examined two of the most affected tissues, the spinal cord and the gastrocnemius muscle.

**Fig. 2:**
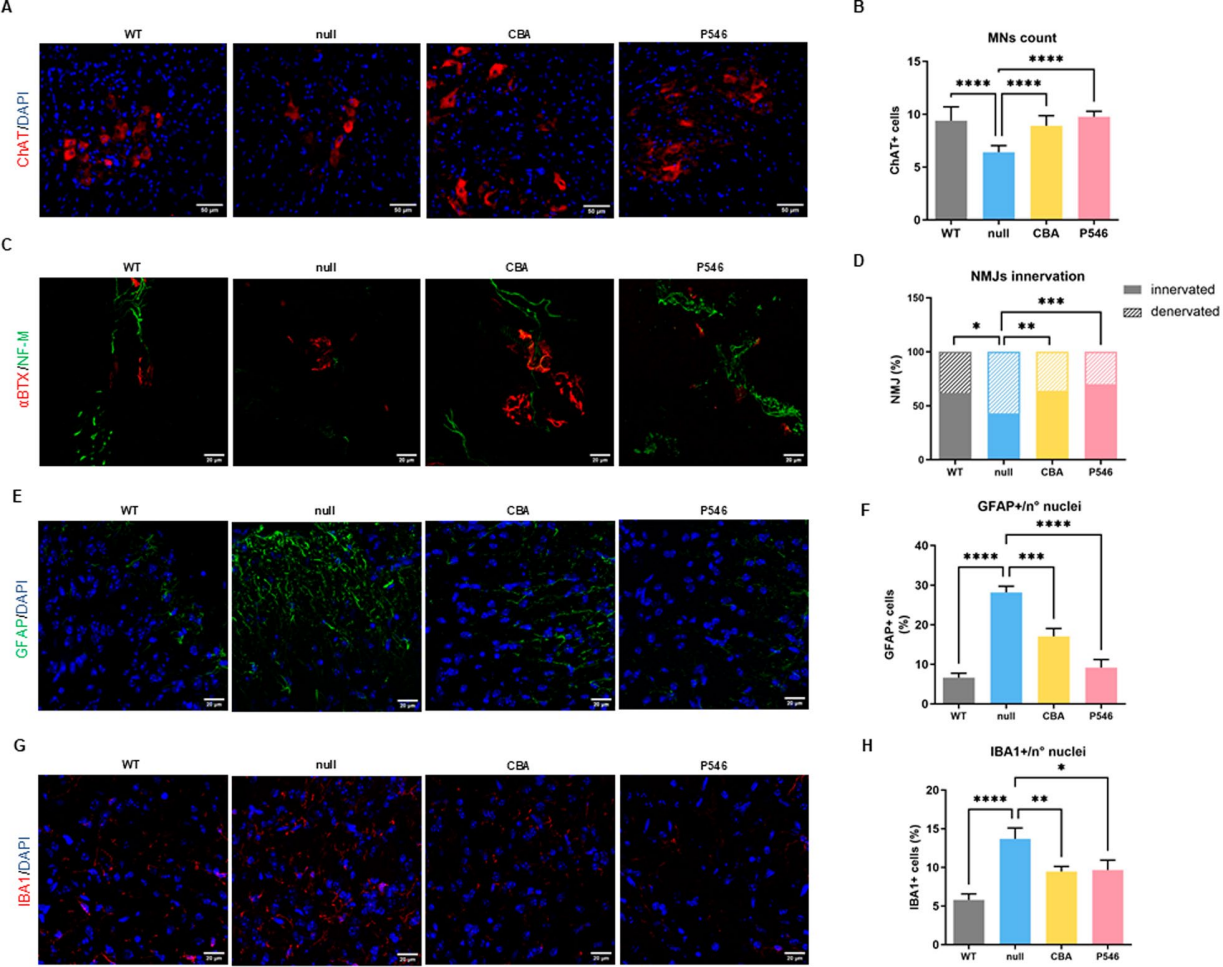
Neuropathological hallmarks rescue: MNs count and NMJs innervation. **A** Representative immunofluorescence images of 20 μm spinal cord’s ventral horn sections from P20 WT and *nmd* treated mice stained for ChAT (red) and DAPI (blue). Scale bar = 50 µm. **B** Average MN number in the lumbar spinal cord (n = 3 animals/group) counted as ChAT-positive cells. **C** Representative immunofluorescence images of 20 μm gastrocnemius sections of WT and *nmd* treated mice stained for NF-M (green) and α-BTX (red) and. Scale bar = 20 µm. **D** Stacked bar plot of NMJs percentage innervation (n = 3 animals/group). **E**, **G** Representative immunofluorescence images of 20 μm spinal cord sections of P20 WT and *nmd* treated mice stained for GFAP (green) (**E**) or IBA1 (red) (**G**) and DAPI (blue). Scale bar = 20 μm. **F**, **H** Respective quantification is represented as the percentage of GFAP **F** or IBA1 **H** positive nuclei divided for the total number of cells (n = 3 animals/group). The values are represented as mean value ± SEM. Statistical significance was determined by one-way ANOVA followed by Dunnett’s multiple comparison test, versus the null group or Contingency test followed by Fisher’s exact test **D**. ****P < 0.0001, ***P < 0.001, **P < 0.01, *P < 0.05

MN count in the ventral horn at P20 revealed a stark decrease of MN number in *nmd* mice treated with the empty vector (6.4 ± 0.6 MNs) compared to their WT counterparts (9.3 ± 1.3 MNs), as expected [30] (Fig. 2A, B, WT vs null *P* < 0.0001, Figure S4A). Treatment with CBA (8.9 ± 0.9 MNs) and P546 (9.7 ± 0.5 MNs) vectors significantly preserved MN numbers, demonstrating a neuroprotective effect in both the cases (Fig. 2A, B, CBA and P546 vs null both *P* < 0.0001, Figure S4A).

Another hallmark of SMARD1 pathology is the impaired neuromuscular junction (NMJ) innervation, which leads to muscle atrophy and paralysis [30]. In our study, null-treated *nmd* mice displayed severe NMJ degeneration, with an average of only 43.4% innervated NMJs vs 62.2% of WT mice (Fig. 2C, D, WT vs null *P* = 0.0106). Instead, both CBA (CBA vs null *P* = 0.0045) and, more effectively, P546 treatments (P546 vs null *P* = 0.0002) considerably improved NMJ innervation, respectively to 63.5% and 69.9% (Fig. 2C, D).

Overall, we demonstrated that gene therapy is able to improve the two main pathological hallmarks of motor neuron diseases.

### Effect of gene therapy on neuroinflammation in the CNS and in muscular compartment

Neuroinflammation, a prevalent pathological characteristic linked to neurodegeneration, has been observed in other MNDs such as SMA or ALS [31]. To unravel this aspect also in the SMARD1 mouse model, for which there is a lack of data, we conducted neuropathological analyses to quantify the neuroinflammatory state in the spinal cords and muscles of the treated mice compared to WT.

Null-treated *nmd* mice spinal cord exhibited pronounced astrogliosis, with glial fibrillary acidic protein (GFAP)-positive cells representing 28.2% ± 1.6 of the total cell population, a stark increase from the 6.6% ± 1.1 observed in WT mice (Fig. 2E, F, WT vs null *P* < 0.0001, Figure S5A). Gene therapy administration provided a reduction of astrogliosis, with either CBA to 17.1% ± 2.0 (CBA vs null *P* = 0.0001) and, more relevantly, with P546 to 9.2% ± 2.0 (Fig. 2E, F, P546 vs null *P* < 0.0001, Figure S5A). Similarly, microglial activation, revealed by ionized calcium-binding adapter molecule 1 (IBA1) expression, was significantly increased in null-treated *nmd* mice at 13.7% ± 1.4 compared to 5.8% ± 0.8 in WT mice (Fig. 2G, H, WT vs null *P* < 0.0001, Figure S5B). This activation was attenuated following treatment with both CBA (9.5% ± 0.7, CBA vs null *P* = 0.0098) and P546 vectors (9.6% ± 1.3, P546 vs null *P* = 0.0255), suggesting a beneficial effect of the gene therapies in moderating microglial activation (Figs. 2G, H and S5B). Overall, these results confirmed for the first time the presence of astrocytosis and microglial abnormal activation in the *nmd* mouse model, similar to other MNDs, and the efficacy of AAV9-based gene therapy in recovering this dysregulation.

**Fig. 3:**
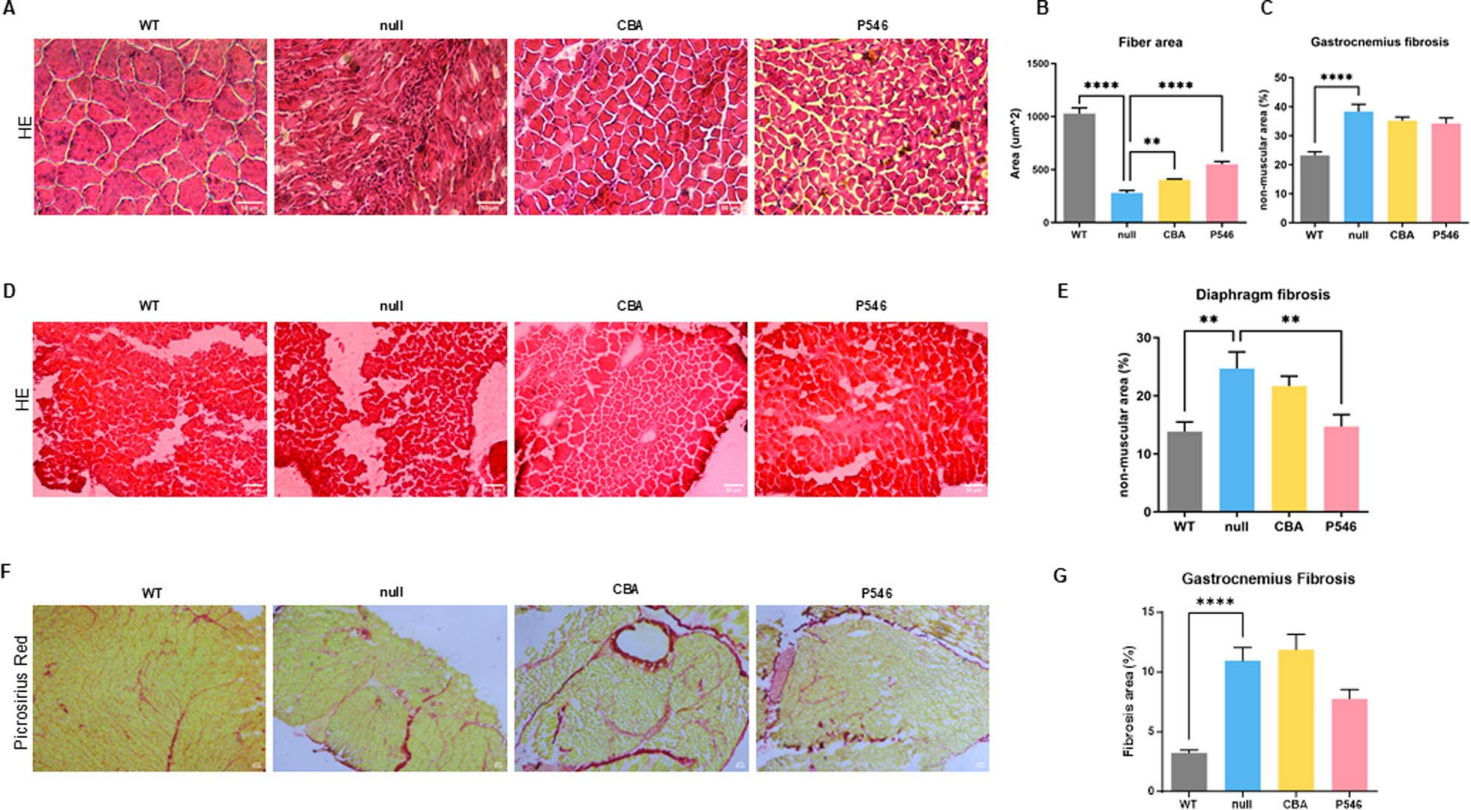
Spinal cord astrocyte gliosis, microglial activation, and muscular inflammatory infiltrate. Representative images of gastrocnemius (**A**) and diaphragm (**D**) muscle fibers of P20 WT and *nmd* treated animals stained with HE. Scale bar = 50 μm. Gastrocnemius fibers CSA quantification expressed in μm^2 (**B**) and relative quantification of fibrotic and necrotic tissue expressed as non-muscular area percentage for gastrocnemius (**C**) and diaphragm (**E**) (n = 3 animals/group). **F** Representative gastrocnemius histological images of P20 WT and *nmd* treated animals stained with picrosirius red. Scale bar = 50 μm. **G** Quantification of the collagen red-stained area in gastrocnemius at P20 on the total surface of the section (%) (n = 3 animals/group). The values are represented as mean value ± SEM. Statistical analysis was performed one-way ANOVA followed by Dunnett’s multiple comparison test, versus the null group. ****P < 0.0001, ***P < 0.001, **P < 0.01, *P < 0.05

Following the results obtained in the CNS compartment, we assessed the peripheral degeneration state. The muscular analysis further revealed that the denervation present in the null-treated mice was associated with a reduced cross-sectional area (CSA) of gastrocnemius muscle fibers (Fig. 3A, B, WT vs null *P* < 0.0001); and an increased fibrotic and necrotic area (Fig. 3A, C, WT 23.2% ± 1.3 vs null 38.3% ± 2.5 *P* < 0.0001) with respect to WT littermates. Remarkably, CBA and P546 vector administrations ameliorated these myopathic changes, significantly improving fiber distribution and size (Fig. 3A, B, CBA vs null* P* = 0.0018; P546 vs null *P* < 0.0001) and slightly decreasing fibrosis (Fig. 3A, C, CBA: 35.2% ± 1.2, P546: 34.2% ± 2.0). In line with the NMJs innervation results, both the constructs resulted beneficial for the muscular compartment, otherwise, the effect mediated by the P546 promoter was more efficient in ameliorating the pathological phenotype in terms of fiber dimension. A more pronounced symptomatology was observed in the diaphragm (Fig. 3D, E), a tissue heavily affected by SMARD1 pathology. The degree of fibrosis was notably elevated in null-treated *nmd* mice (Fig. 3D, E, WT vs null *P* = 0.0013), but showed important improvement following treatment with both constructs, significant only with the P546-based vector (Fig. 3D, E, P546 vs null *P* = 0.0096). To deepen fibrosis analysis, Picro Sirius Red staining on gastrocnemius muscle was performed revealing a marked increase in null*-nmd* mice compared to wild-type mice (Fig. 3F, G, WT vs null *P* < 0.0001). In the CBA-treated group, fibrosis levels remained comparable to those observed in the null*-nmd* mice. Conversely, in the P546-treated group, there was a clear trend toward reduced fibrosis; notably, the difference between wild-type and P546 was no longer statistically significant, indicating a recovery of fibrosis levels toward those of wild-type mice (Fig. 3F, G).

### Gene therapy long term effect on CNS in adult mice

To assess long term effects of gene therapy, we analyzed the hallmarks of the pathology later in life at P200 in *nmd*-treated mice compared to WT. The null*-*treated group was not present in the analysis due to their premature death.

**Fig. 4:**
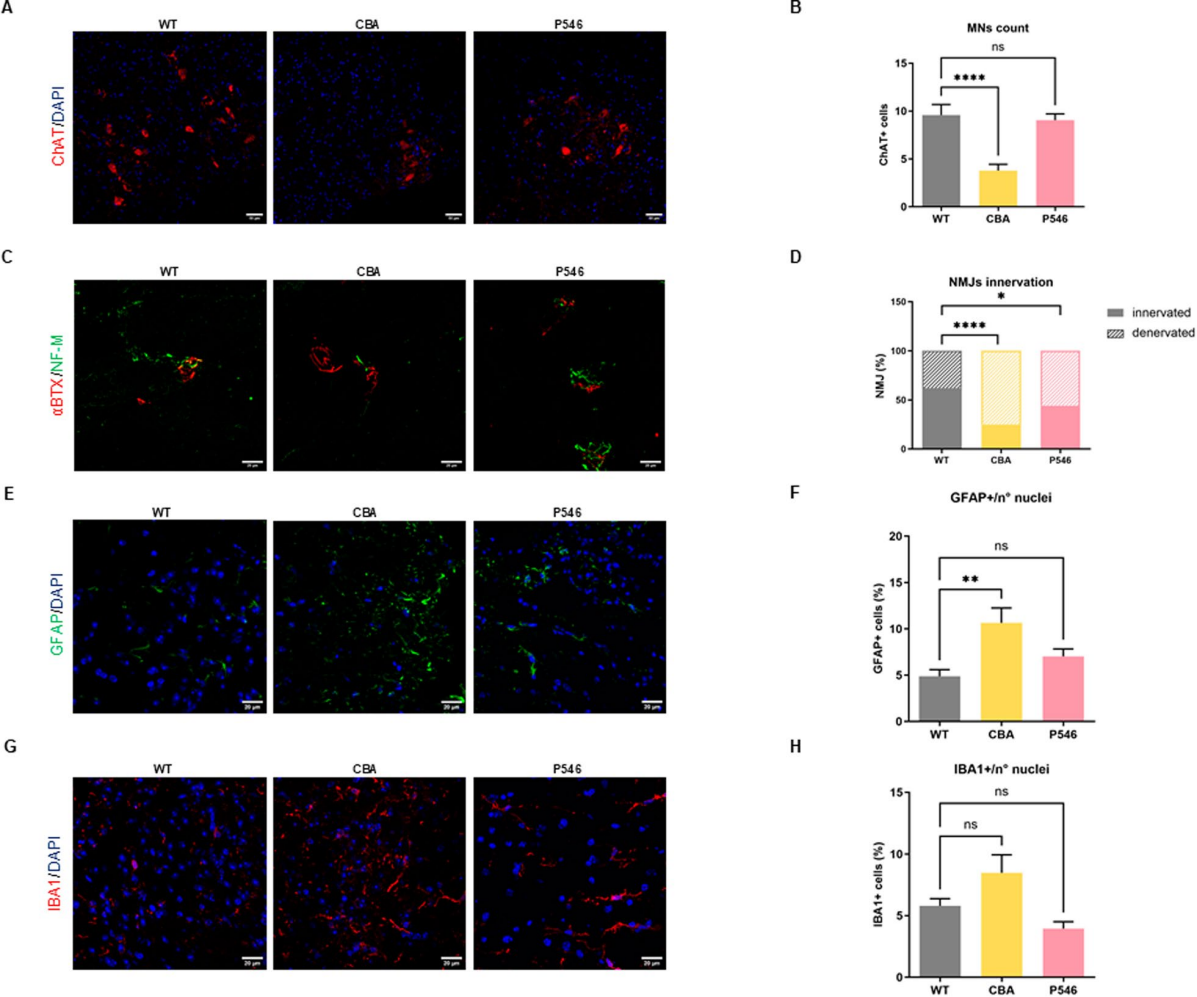
Long-term effects on MN count, NMJ innervation and neuroinflammatory marker in adult mice. **A** Representative immunofluorescence images of 20 μm spinal cord’s ventral horn sections of P200 WT and treated *nmd* mice stained for ChAT enzyme (red) and DAPI (blue). **B** Average MN number in the total lumbar spinal cord (n = 3 animals/group). **C** Representative immunofluorescence images of 20 μm gastrocnemius sections of P200 WT and *nmd* treated mice stained for NF-M (green) and α-BTX (red). Scale bar = 20 µm. **D** Stacked bar plot of NMJs percentage innervation (n = 3 animals/group). Representative immunofluorescence images of 20 μm spinal cord sections of P200 WT and *nmd* treated mice stained for GFAP (green) (**E**) or IBA1 (red) (**G**) and DAPI (blue). Scale bar = 20 μm. Respective quantifications are represented as the percentage of GFAP (**F**) or IBA1 (**H**) positive nuclei divided by the total number of cells (n = 3 animals/group). The values are represented as mean value ± SEM. Statistical significance was determined by one-way ANOVA followed by Tukey’s post hoc test (**B**, **F**, **H**) or Contingency test followed by Fisher’s exact test (**D**). ****P < 0.0001, **P < 0.01, *P < 0.05

First, we confirmed that the amount of the IGHMBP2 protein in the spinal cord was maintained with a trend similar to P20, with higher protein levels in the CBA group and an expression matching endogenous levels more closely with the P546 promoter (Figure S2C,D). Regarding the typical pathological markers of the disease, we observed MNs preservation in the spinal cord only with the P546 construct (9.1 ± 0.7 MNs), with a very similar level to the WT counterpart (9.6 ± 1.1 MNs), while the CBA vector showed much lower maintenance of MNs (3.8 ± 0.7 MNs) (Fig. 4A, B, CBA vs WT *P* < 0.0001, Figure S4B). Similarly, the NMJ innervation was much lower in the CBA-treated mice resulting in only 24.8% innervated NMJs compared to 62.4% of WT animals (Fig. 4C, D, CBA vs WT *P* < 0.0001). In comparison, the P546 construct maintained an improved innervation percentage of 44.2%, while some signs of degeneration were still evident as compared to WT animals (Fig. 4C, D, P546 vs WT *P* = 0.0158).

In terms of neuroinflammation, quantitative analysis revealed that at P200 the CBA group displayed an increased number of GFAP-positive astrocytes compared to WT (Fig. 4E, F, 10.6% ± 1.6 and 4.9% ± 0.7 respectively, CBA vs WT *P* = 0.0011, Figure S5A). In contrast, the astrocytosis detected in the P546 group (7.0% ± 0.8) was not statistically different from that of WT animals (Fig. 4E, F and S5A). Importantly, with both the constructs, levels were still lower than with AAV9-null at P20 (28.2% ± 1.6). In terms of microglial activation, the percentage of IBA1 + cells was 5.7% ± 0.6 for WT, 8.5% ± 1.5 with the CBA treatment and 3.9% ± 0.6 with the P546 promoter, without significant deviations from WT (Figs. 4G, H and S5A).

### Gene therapy long term effect on muscle degeneration in adult mice

**Fig. 5:**
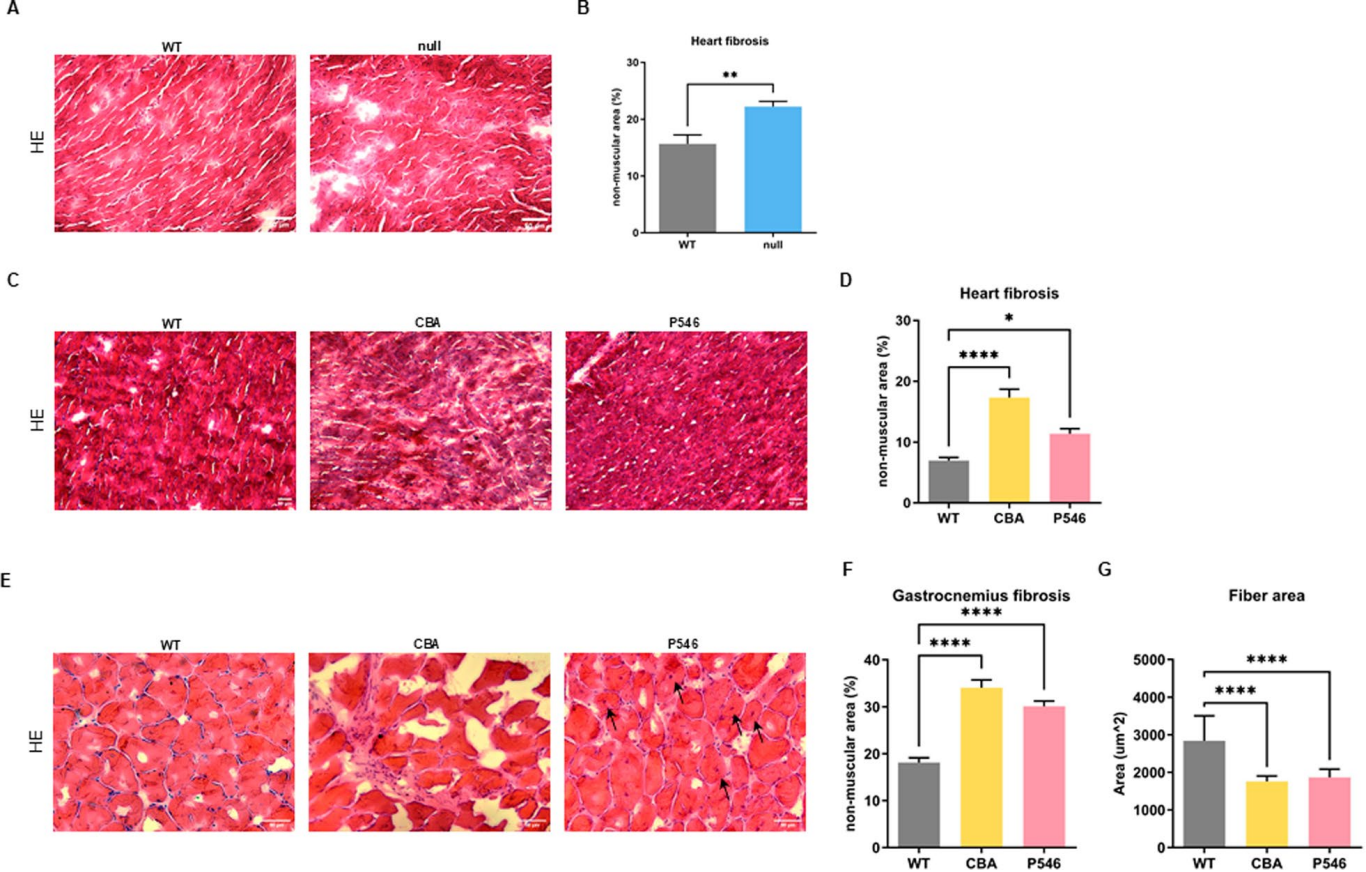
Late-onset cardiac defect rescue in adult mice treated with gene therapy. Representative histological images of 15 μm cardiac muscle fibers of P20 (**A**) and P200 (**C**) WT and *nmd* treated animals stained with HE. Scale bar = 50 μm. **B** Respective quantification of fibrotic and necrotic tissue expressed as non-muscular area percentage in cardiac muscle (n = 3 animals/group). **E** Representative histological images of 15 μm gastrocnemius muscle fibers of P200 WT and *nmd* treated animals stained with HE. Scale bar = 50 μm. **F** Respective relative quantification of fibrotic and necrotic tissue expressed as non-muscular area percentage in gastrocnemius muscle and (**G**) fibers CSA quantification expressed in μm^2 (n = 3 animals/group). Arrows indicate centrally nucleated fibers. The values are represented as mean value ± SEM. Statistical significance was determined by two-tailed, unpaired Student’s t-test followed by Mann Whitney post doc test (**B**) and one-way ANOVA followed by Tukey’s post hoc test (**D**, **F**, **G**). ****P < 0.0001, ***P < 0.001,**P < 0.01, *P < 0.05

Cardiomyopathy and cardiac myocyte defects were previously described in this SMARD1 mouse model in the later stage of the disease, around 4–6 weeks of age [32, 33]. In addition, since *IGHMBP2*-treated adult mice died suddenly without simultaneous clear signs of degeneration and suffering, we hypothesized that cardiac issues could be a potential death causative factor. Therefore, we assessed cardiac conditions in WT and *nmd*-treated adult mice. In our model, we confirmed the presence of an important initial cardiac defect already early in life at P20 with a significant increase in muscle fibrosis with respect to WT animals (Fig. 5A, B, WT 15.6% ± 1.6 vs null 22.2% ± 0.9 *P* = 0.0015). In the animals which are still alive at P200, the level of cardiac fibrosis in the CBA-treated group was more than double the level of WT animals (Fig. 5C, D, CBA 17.3% ± 1.4 vs WT 6.9% ± 0.5 *P* < 0.0001), while this defect was mitigated in the P546 group, even if signs of cardiac fibrosis were present (Fig. 5C, D, P546 11.4% ± 0.9 vs WT *P* = 0.0321).

In parallel, we conducted further HE analysis on gastrocnemius muscles also at P200 (Fig. 5E–G). In line with findings at earlier stage and with cardiac results, we observed the presence of muscular degeneration in the hindlimbs, in particular (i) increased non-muscular area percentage in both the treated groups, (Fig. 5E, F, CBA 34.0% ± 1.7 and P546 30.1% ± 1.1 vs WT 18.1% ± 1.0 *P* < 0.0001) and (ii) reduced fibers dimension (Fig. 5E, G, CBA and P546 vs WT *P* < 0.0001). For both markers, the P546 cohort had better average values with respect to the CBA group, even if not statistically different. To be noted, the values were comparable to the one obtained at P20, suggesting that the effect is stable over time. Interestingly, the analysis of gastrocnemius muscles in adult animals, highlighted the presence of centrally nucleated fibers, sign of fiber regeneration, in the P546 and not in the CBA group, supporting the more sustained effect at this later stage of the pathology with the P546-based construct [34]. Overall, these data confirmed that the muscular degeneration, particularly the cardiac defect of the SMARD1 mouse model, is already present in the early phases of the disease, in terms of fibrosis and functionality, and could be partially rescued with gene therapy.

### Gene therapy with the P546 vector attenuation of neuroinflammation-related gene changes in SMARD1 mouse model spinal cord

To investigate the molecular mechanisms responsible for the therapeutic effects of gene therapy, we conducted RNA sequencing analysis on spinal cords of P20 mice, for the first time comparing WT, null-treated and treated with P546 vector, that has shown more safety and long term efficacy.

**Fig. 6:**
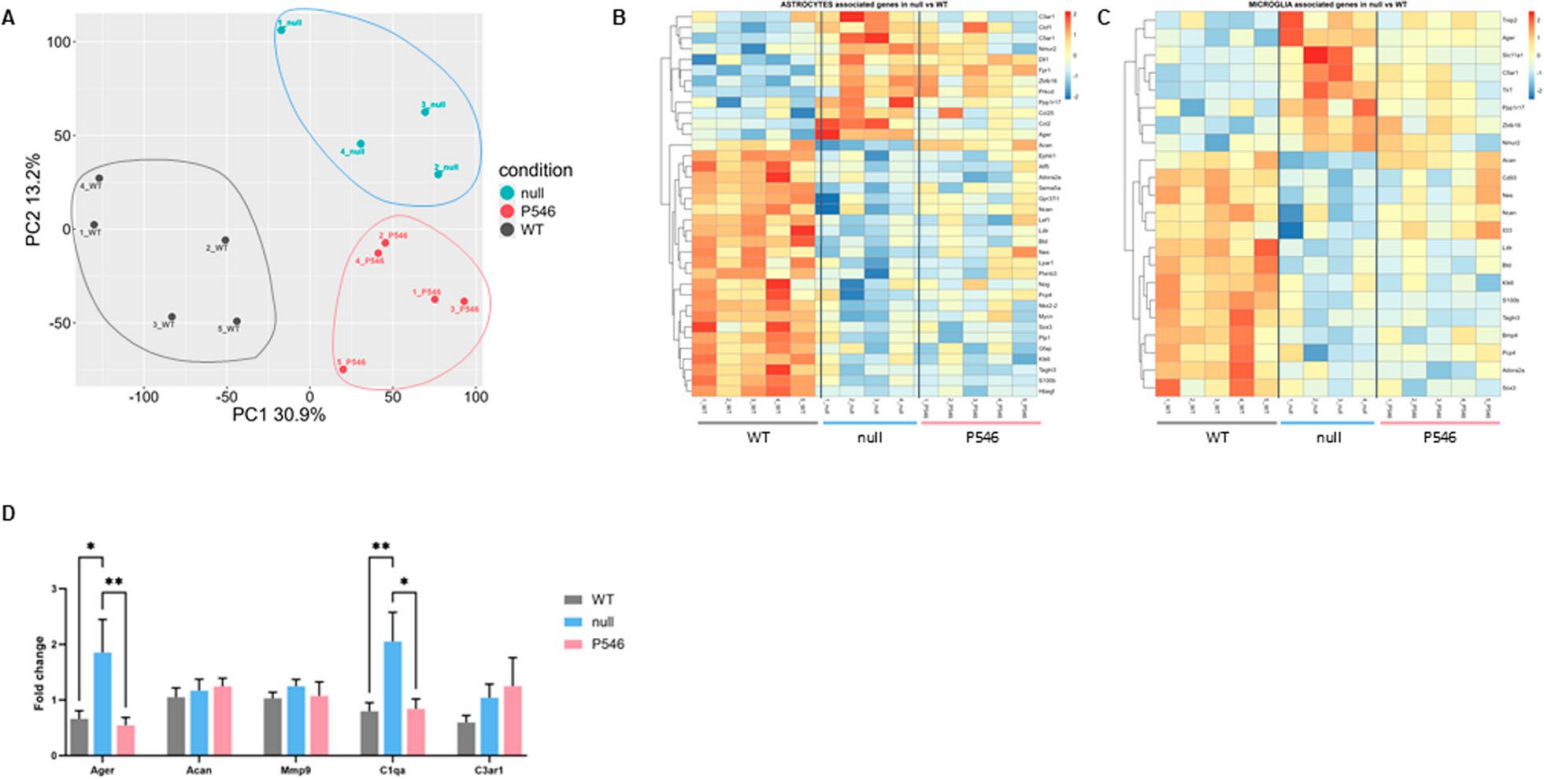
Spinal cord RNAseq analysis. **A** PCA of RNA sequencing bulk analysis on WT, *nmd-*null and *nmd*-P546 spinal cord at P20 (n = 5 animals for WT and P546 and n = 4 animals for null group). **B**–**C** Heatmap of astrocyte- (**B**) and microglia-related genes expression in the different groups. The comparison was made with genes with padj < 0.05 and logFC > 0.5 or < -0.5. **D** mRNA levels detected by qPCR of genes selected from RNA sequencing bulk analysis in WT, *nmd-*null and *nmd*-P546 treated spinal cord. Statistical significance was determined by one-way ANOVA followed by Dunnett’s multiple comparison test, versus the null group. **P < 0.01, *P < 0.05

Principal Component Analysis (PCA) distinctly segregated the three cohorts, confirming the unique transcriptional profiles for each group (Fig. 6A). In null-treated *nmd* mice, we identified 549 genes with increased expression and 323 with decreased expression compared to WT controls (Figure S7A). Among upregulated genes, Gene Ontology enrichment highlighted those involved in immune system activation, especially genes linked cytokines-mediated pathways (Figure S7D), whereas the downregulated genes were linked mainly to muscle development, differentiation and contraction, and lipid metabolism (Figure S7D).

A deeper analysis on astrocyte (Fig. 6B) and microglia (Fig. 6C) associated genes (see Material and Methods section) was assessed observing a clear dysregulation between WT and null-treated animals, confirming the presence of neuroinflammation in SMARD1. Interestingly, regarding astrocytes, 28 genes were differentially expressed: the upregulated genes were involved in inflammation and in astrocyte response to stress or degenerative disease, while the downregulated ones belonged to astrocytes differentiation pathway. Moreover 19 microglia associated genes were differentially expressed: the upregulated genes had mainly a role in microglia activation while those downregulated were linked to response and differentiation.

Comparing P546-treated *nmd* mice to their null counterparts, we found the 38 upregulated genes predominantly involved in monocarboxylic/organic acid metabolism processes, and 53 downregulated genes mainly associated with immune response processes and response to cytokines (Figure S7B,D). Data suggested that the P546 treatment could mitigate the immune system activation present in the CNS of null-*nmd* mice. Notably, focusing on astrocyte and microglia associated genes, involved in neuroinflammation, the treatment was able to significantly normalize the expression of some of them, namely *Aggrecan (Acan), Complement C3a receptor 1 (C3ar1), Advanced glycosylation end-product specific receptor (Ager),* and *Complement C1q A chain (C1qa)* for astrocytes and *Acan, Interleukin-31 (Il33), Matrix m9 (Mmp9), Ager, Solute carrier family 11 member 1 (Slc11a1)*, *C1qa* and *Transmembrane immune signaling adaptor TYROBP (Tyrobp)* for microglia (Fig. 6B, C). qPCR validation performed on these genes confirmed alteration in null-nmd mice vs WT and also the significative rescue after treatment for *Ager* and *C1qa* (Fig. 6D, *Ager*: WT vs null *P* < 0.05, null vs P546 *P* < 0.01, *C1qa:* WT vs null *P* < 0.01, null vs P546 *P* < 0.05).

## Discussion

The present study marks a pivotal advance in gene therapy targeting SMARD1, a rare fatal genetic disorder caused by mutations in *IGHMBP2* gene, lacking approved efficacious treatments. Recently, gene therapy has obtained promising results in the treatment of monogenic disorders especially for neurological diseases. One big step forward in the field was the approval of the first AAV gene therapy treatment (Zolgensma^®^) for the classical 5q SMA form, in 2019 by FDA and in 2020 by EMA [17, 18]. The similarity between the two muscular atrophies led to the hypothesis that the approach could be beneficial also for SMARD1. Our previous study firstly demonstrated that the presymptomatic intravenous delivery of wild-type *IGHMBP2*, the causative gene of SMARD1, by AAV9, rescued disease phenotype in the same *nmd* mouse model [23]. Efficacy of AAV gene therapy was further confirmed in other SMARD1 mouse models by Lorson et al. [35], underlying the promise of the approach.

Our present study supported lead candidate selection for IND-enabling studies that ultimately lead to the initiation of the first in human clinical trial for treatment of SMARD1/CMT2S [25] (NCT05152823). We compared safety and efficacy of two AAV9 viral vectors in a blinded manner to support promoter selection. Of note, both promoters used in this study were already used in clinical settings: the Chicken β-Actin promoter (CBA) [20, 28] and the truncated methyl-CpG-binding protein 2 (MeCP2) promoter (P546) [29], that has been proved to assure long lasting and physiological transgene expression in Rett syndrome [27] and in Batten disease preclinical models [26].

Differently from our previous study [23], the treatment was performed presymptomatically by a local intracerebroventricular (ICV) injection, a route chosen for its superior efficacy and additional advantages including reduced systemic dispersion, potentially lower required dosage, decreased cost, and minimized immunogenicity and toxicity—all essential considerations for clinical translation [21, 25, 26]. Our results confirmed an important increase of the IGHMBP2 protein expression in the spinal cord with both constructs; however, CBA promoter led to a protein expression significantly higher than WT levels in the CNS at P20, whereas the P546 promoter restored more physiological levels of expression, a trend that was also maintained at later stages (P200).

Both vectors lead to a highly significant extension of survival and amelioration of the motor phenotype if compared to the empty vector (null), confirming the efficacy of gene therapy in the SMARD1 mice.

No signs of toxicity have been detected, hepatic and renal biological markers analyzed in mice serum did not result altered after the treatment, a cardinal endpoint for the translational perspective. An early mortality was observed in a small group of CBA treated animals around P20-40. Excessive transgene overexpression has been associated with potential toxicity through unknown mechanisms [20]. However, given the relatively low dose used and the limited number of early deaths observed, we hypothesize that the cause is more likely related to an insufficient rescue of disease pathology outside the CNS—such as in the heart.

Strikingly, both constructs led to a substantial amelioration of pathological hallmarks. Improvements were observed in the CNS, including an increased number of MNs and a reduction in neuroinflammation: in muscles, treatment resulted in enhanced NMJ innervation, increased muscle fiber diameter, and reduced fibrosis.

The alteration of astrocytes and microglia status in neurodegenerative diseases has long been recognized, but it has never been assessed in SMARD1. Since the involvement of astrocyte and microglia activation in SMARD1 disease is not well understood, in recent years in vitro (patients’ derived cells) and in vivo (transgenic mouse) models have been used to study their functional or pathological role in different neurodegenerative disorders including ALS. Microglial gene signature transition from homeostatic to an inflammatory state accelerates pathology progression and neurodegeneration in ALS through multiple mechanisms [36], while astrocyte reaction appears at the early stages of ALS and is probably directly involved in the degeneration of MNs [37]. Our data demonstrated for the first time the involvement of neuroinflammation in the SMARD1 model, confirmed by the presence of increased GFAP + and IBA1 + cells in *nmd* spinal cords. Notably, gene therapy reduced astroglial and microglial activation providing additional evidence of inflammatory role and efficacy of the therapeutic strategy. However, further studies are required to define the precise relationship between inflammation and motor neuron degeneration.

RNA sequencing performed on the spinal cord of WT, null, and P546 treated animals, other than investigating the general molecular mechanisms underlying the observed phenotypic improvements, confirmed the presence of activated microglia and astrocytes in SMARD1 and highlighted the positive impact of gene therapy. First, regarding the general molecular alteration, we observed un upregulation of the expression of genes associated with immune response in null-treated *nmd* mice compared to WT (e.g. *Oas1g, Ccl5, Cxcl10, Bst2, Ifit3, Ifi204*). The altered expression of immune response related genes was restored after P546 treatment suggesting an immunomodulatory effect of the gene therapy that could be pivotal in treating neurodegenerative conditions such as SMARD1, where immune activation exacerbates disease progression. On the other hand, null-treated animals presented a downregulation of genes involved in lipid metabolism vs WT (e.g. *Cyp1a2, Serpina6, Fabp1, Fitm1, Prkag3, Bglap, Alox12*), confirming the potential involvement of energetic metabolism impairments in SMARD1, similar to what is already suggested in other neurodegenerative diseases such as Alzheimer and Parkinson Diseases, ALS and Huntington's Disease [38]. We further analyzed RNA seq data focusing on astrocytes and microglia associated genes, selected based on GO:BP datasets (see Material and methods section), confirming dysregulation in both groups in null animals vs WT. Interestingly, P456 treatment restored the expression of some deregulated genes, in particular those linked to microglial and astrocytes activation (*Acan*, *Ager, C3ar1, C1qa, Slc11a1, Tyrobp, Mmp9*). It has already been observed that the deletion of *C1qa* and *C3* genes mitigated microglial toxicity and rescued transactive response DNA binding protein 43 (TDP-43) proteinopathy and neurodegeneration in mice with microglial activation mediated by progranulin deletion (*Grn*^−/−^ mouse model) [39]. Among restored genes we found *Matrix metalloproteinase-9* (*Mmp9*). MMP9 released by glial cells may also contribute to the pathogenesis of ALS, leading to MN degeneration and muscle atrophy, resulting in alterations in the extracellular matrix [39] and a neurotoxic role for MMP9 has been already demonstrated using SOD1 and TDP-43 animal models for ALS [40, 41]. Another restored gene was *Acan*, which is widely known to be upregulated after acute injuries like those of the spinal cord. While its role in progressive degeneration is less defined, increased expression has been observed in rats with retinal degeneration [42].

It is well known that the *nmd* mouse model develops dilated cardiomyopathy, even when IGHMBP2 expression is selectively rescued in neurons only [43]. Moreover, the absence of reported cardiomyopathy in SMARD1 patients does not exclude the possibility of undetected cardiac complications masked by the patients premature death [44]. Our data suggest that cardiac involvement in SMARD1 could play a role in the pathology and should be taken into account when patients receive future treatments that extend their lifespan. Since, in our hands, treated adult mice often died suddenly without clear signs of neuromuscular degeneration, and the alterations observed in the heart tissue of null-treated mice were only partially rescued by gene therapy, we hypothesize that death is more likely caused primarily by cardiac defects—potentially exacerbated by the increased mobility of treated mice due to MN rescue—rather than by MN pathology. However, further research is needed to fully understand the extent of cardiac involvement in SMARD1 and to determine the specific causes and underlying mechanisms of the complications that may emerge after gene therapy.

We analyzed the long-term beneficial effects of gene therapy in adult mice (P200) and observed that, even though IGHMBP2 expression is maintained in the spinal cord with both vectors, the therapeutic potential of P546 is clearly more pronounced in both the CNS and muscle tissues. Indeed, it proved to be significantly more effective than CBA in preserving MN numbers and NMJ innervation, while also reducing neuroinflammation and mitigating cardiac defects at P200.

Overall, while both gene therapy constructs highly significantly improved survival and histological phenotypes, the P546 promoter outperformed the CBA promoter likely due to its ability to allow more even expression in multiple cell types such as neurons and glia [45] and to drive the expression of the gene of interest in a physiological manner. This hypothesis is supported by a recent study that demonstrated, in another monogenic disease, that restoring SMN expression close to physiological levels using AAV9-mediated gene therapy improved safety and efficacy in a SMA mouse model compared with uncontrolled high-level expression [46]. Moreover, P546 construct has superior efficacy in the late stages of the disease also in neuroinflammation and cardiac pathology. Collectively, these findings support the safety profile and sustainable therapeutic efficacy of P546 promoter construct.

## Conclusions

In summary, our results underscore the significant contribution of astrogliosis and microgliosis to SMARD1 pathology and demonstrate that the ICV gene therapy approach can effectively rescue these pathological aspects, in addition to those specifically associated with motor neuron dysfunction, and highlight the therapeutic potential of P546-AAV9 gene therapy for SMARD1 treatment.

## Materials and methods

### Murine model

B6.BKS-*Ighmbp2*^*nmd2J*^/J animals were purchased from The Jackson Laboratory (Bar Harbor, ME), catalog #002521 [47]. All animal procedures received approval from the Institutional Animal Care and Use Committee (IACUC) of the University of Milan and the Italian Ministry of Health, adhering to national guidelines (D.I. no. 116, G.U. supplement 40, February 18th, 1992; Circular no. 8, G.U., July 14th, 1994) under the approved protocol 707/2019-PR. Heterozygous mice carrying the *Ighmbp2* mutation were crossbred to generate offspring with homozygous mutation (*nmd*^*2J*^/*nmd*^*2J*^, hereafter referred to as *nmd*) while wild-type (WT) littermates served as healthy controls.

Genotyping was performed using DNA from tail biopsies collected from newborn pups, following a modified protocol from the Jackson Laboratory (forward primer: 5′-CCT GAT TTT GGC TCT GGT CC-3′; reverse primer: 5′-GCT CCT GAT GAT CCA ATG GT-3′). The homozygous presence of the mutated allele, which was selectively cleaved by the DdeI enzyme, indicated affected animals. Genotyping classified animals as either WT, heterozygous, or homozygous for *Ighmbp2* mutation.

### Viral vectors structure and administration

Three AAV9 vectors provided by Nationwide Children’s Hospital (Columbus, OH) were used. Two constructs, packaged into either single-strand AAV9 genome, contained human *IGHMBP2* cDNA driven by two different promoters: the CMV-enhancer Chicken β-Actin (CBA) and a synthetic truncated version of the methyl-CpG-binding protein 2 (MeCP2) promoter, called P546 (Figure S1). The third vector, indicated ad AAV9-null, was an empty viral particle and served as a negative control in the short-term examination. Mice homozygous for *Ighmbp2* mutation received randomly a single intracerebroventricular (ICV) injection at P1 with 5 × 10^10^ vg/animal of ssAAV9-CBA::*IGHMBP2* (n = 28), ssAAV9-P546::*IGHMBP2* (n = 28), or AAV9-null virus (n = 28), following a method outlined in previous literature [48]. The investigator administering the injection was blinded to the promoter types. WT littermates (n = 28) were included as controls, and *nmd* mice treated with empty null virus served as a pathological baseline (negative control). Female and male mice were equally distributed among all the experimental groups.

### Phenotype and survival analysis

Each animal was weekly monitored for morbidity and mortality; the end of the study was set at 300 days of life. Starting from 2 weeks of age, body weight and behavioral motor responses were weekly recorded. Motor function was assessed using the hindlimb splay test, which evaluates the mice’s ability to open their hindlimbs when suspended head-down by the tail. This test was classified on a 5-step scale from 0 to 4. Rotarod performance test (Rotarod 7650, Ugo Basile, Varese, Italy) was firstly performed at P45 and conducted monthly to assess the mice’s motor ability and balance. An accelerating protocol was used, increasing the speed from 4 to 40 rpm over 300 s (with an increase of 1 rpm every ∼8.3 s), and the latency to fall was recorded. The test results were shown until > 3 animals/group were alive.

### Histological and immunohistochemistry analysis

Tissues were collected at P20 (n = 3 animals/group) and at P200 (n = 3 animals/group). Diaphragm, gastrocnemius and cardiac muscles were rapidly frozen on dry ice, while spinal cords were fixed in a 4% paraformaldehyde solution (Sigma-Aldrich, St. Louis, MO). Each tissue was embedded in the Tissue-Tek^®^ Optimal Cutting Temperature (OCT) Compound (Sakura Finetek Europe, Alphen aan den Rijn. Netherlands) and sectioned using a cryostat (Leica, Wetzlar, Germany) into 15 or 20 μm sections on polylysine-coated glass slides (Epredia, Kalamazoo, MI).

#### Immunofluorescence

Primary antibody against choline-acetyltransferase (ChAT, 1:250, MilliporeSigma, Burlington, MA), glial fibrillary acidic protein (GFAP, 1:600, MilliporeSigma, Burlington, MA), ionized calcium-binding adaptor molecule 1 (IBA1, 1:500, Wako, Neuss, Germany), neurofilament medium chain (NF-M, 1:250, MilliporeSigma, Burlington, MA) and α-bungarotoxin conjugated with Alexa 555 (α-BTX, 1:200, Invitrogen, Waltham, MA) were used. Images were acquired using a Leica SP8 confocal microscope (Leica, Wetzlar, Germany) at various magnifications.

Motor neuron (MN) counts were conducted on 30 spinal cord serial sections, each taken at least 100 μm apart, by quantifying the mean number of ChAT-positive MNs in the ventral horns of the lumbar spinal cord (L1-L5) [49]. Astrocyte gliosis and microglia activation were evaluated in the mice’s spinal cord by determining the percentage of cells surrounded by more than 50% of their perimeter by GFAP or IBA1 signal [50]. The total number of neuromuscular junctions (NMJs) and the percentage of innervated NMJs, identified by the colocalization of NF-M and α-BTX signals, were counted (n = 100 junctions/animal) [51].

#### Histology

Diaphragm, gastrocnemius and cardiac muscle slides were stained with hematoxylin–eosin (HE). Images were captured at 10X or 20X magnification using an optical microscope Leica DMi8 (Leica, Wetzlar, Germany). The cross-sectional area (CSA) of each fiber was determined by manually tracing their perimeters using LAS software version 4.9.0 (n = 600 muscular fibers/animal) [52]. The fibrotic and necrotic percentage of each image was calculated by using the HE colors deconvolution plugin of ImageJ software [53].

Picro Sirius Red Kit (Abcam) was used to identify muscle fibrosis. Briefly, gastrocnemius slides were fixed in Bouin's solution for 15’ at 56 °C and then stained using Picro Sirius Red Kit following manufacturer's instructions. The area of collagen fibers in respect with the total slice have been quantified by using the colors deconvolution plugin of ImageJ software [53].

### RNA isolation and RNAseq analysis

RNAseq bulk analysis was performed on spinal cord-extracted RNA by BGI Genomics (Guangdong, China). Batch correction was applied to the raw count with Combat_seq function of R SVA package, and principal component analysis (PCA) was conducted using the prcomp function from the R stats package. To evaluate inflammation observed by histological analysis, a list of genes associated with microglia and astrocytes was generated by selecting genes involved in pathways related to these cell types in the GO:BP datasets. The comparison was made with genes that had padj < 0.05 and logFC > 0.5 (upregulated) or logFC < -0.5 (downregulated).

### qPCR analysis

cDNA for real-time PCR experiments was synthesized from 1.5 μg of total RNA, using the Ready-To-Go kit (GE Healthcare). The expression levels of *Acan* (Mm00545794_m1, Thermo Fisher Scientific)*, C3ar1* (Mm02620006_s1, Thermo Fisher Scientific*), Ager* (Mm01134790_g1, Thermo Fisher Scientific), *C1qa* (Mm07295529_m1, Thermo Fisher Scientific) and *Mmp9* (Mm00442991_m1, Thermo Fisher Scientific) were assessed by quantitative analysis on a 7500 Real-Time PCR System (Applied Biosystems). Data were normalized to the average levels of *B-Actin* (Mm00607939_s1, Thermo Fisher Scientific).

### Statistical analysis

Statistical analyses were performed using GraphPad Prism^®^ version 10.0 (GraphPad Software Inc.). Survival data were represented using Kaplan–Meier curves and analyzed with the Log-rank (Mantel-Cox) test. Other data were expressed as mean ± standard error of the mean (SEM). Each data group was normalized to the mean of the control group (WT). One-way analysis of variance (ANOVA) was used for multi-comparison analysis followed by Tukey’s post hoc test or Dunnett’s multiple comparisons test. Two-tailed, unpaired Student’s t-test followed by Mann Whitney test was used to compare two different groups. The NMJ innervation was analyzed by the Contingency test followed by Fisher’s exact test. A *P*-value < 0.05 was considered statistically significant for all analyses.

## Supplementary Information

Supplementary material 1

Supplementary material 2

## Data Availability

RNAseq dataset will be uploaded on Gene Expression Omnibus repository.
