# Supplementary material for: AAV9 gene therapy optimization for SMARD1/CMT2S: safety and long-term efficacy comparison of two vectors in a SMARD1 preclinical model
Source: J Biomed Sci. 2026 Jan 4;33:1. doi: 10.1186/s12929-025-01204-z (PMC12765291; doi:10.1186/s12929-025-01204-z)
Supplement: Supplementary file 1 — Supplementary material 1 [file 12929_2025_1204_MOESM1_ESM.pdf]

Supp. Materials and Methods

Western Blot analysis

Proteins were extracted from the spinal cords of mice injected at P1 and then euthanized at P20 (n=3 animals/group) and P200 (n=3 animals/group). A quantity of 20 µg of protein per sample was resolved on a NuPAGE Bis-Tris gradient polyacrylamide gel (Invitrogen, Waltham, MA) and then electrophoretically transferred to a nitrocellulose membrane (GE HealthCare, Chicago, IL). The target protein, human IGHMBP2, was detected with an anti-human IGHMBP2 antibody (1:800 dilution) (MilliporeSigma, Burlington, MA) and quantified relative to α-actin (1:1’000 dilution) (Sigma-Aldrich, St. Louis, MO) as a loading control.

Hematological analysis

The hematological markers were measured from serum of P20 mice (n=4 animals/group) obtained as previously described[44]. Serum samples were analyzed by Charles River Laboratories (Lodi, Italy) for various biomarkers, including alanine transaminase (ALT), alkaline phosphatase (ALP), creatine kinase (CK), lactate dehydrogenase (LDH), total bilirubin, glucose, creatinine, urea, albumin, total proteins, and total cholesterol.

RNAseq Volcano Plot and GSEA analysis

After batch correction, differential expression analysis was performed with DESeq2 and the differentially expressed genes were visualized as a Volcano plot using gglot2[49]. Genes with padj<0.05 and logFC>1 were considered upregulated, while those with padj<0.05 and logFC<-1 were considered downregulated. Gene ontology (GO) enrichment analysis was performed on these genes with GSEA (fgsea package in R), using Gene Ontology Molecular Function dataset from MSigDB as reference. The most relevant pathways, selected with a percentage threshold (> 95%) that determines the relevance of a pathway according to its size, were represented as a bubble plot created with ggplot2.

**Supp.Table 1. Astrocyte- and microglia-related pathways**	**GO:BP dataset**
Astrocyte activation (GO:0048143)	GO_Biological_Process_2023
Astrocyte activation (GO:0048143)	GO_Biological_Process_2017b
Astrocyte activation (GO:0048143)	GO_Biological_Process_2018
Astrocyte chemotaxis (GO:0035700)	GO_Biological_Process_2017b
Astrocyte development (GO:0014002)	GO_Biological_Process_2023
Astrocyte development (GO:0014002)	GO_Biological_Process_2017b
Astrocyte development (GO:0014002)	GO_Biological_Process_2018
Astrocyte development (GO:0014002)	GO_Biological_Process_2021
Astrocyte development (GO:0014002)	GO_Biological_Process_2015
Astrocyte differentiation (GO:0048708)	GO_Biological_Process_2023
Astrocyte differentiation (GO:0048708)	GO_Biological_Process_2017b
Astrocyte differentiation (GO:0048708)	GO_Biological_Process_2018
Astrocyte differentiation (GO:0048708)	GO_Biological_Process_2021
Astrocyte differentiation (GO:0048708)	GO_Biological_Process_2015
Negative regulation of astrocyte differentiation (GO:0048712)	GO_Biological_Process_2023
Negative regulation of astrocyte differentiation (GO:0048712)	GO_Biological_Process_2021
Negative regulation of astrocyte differentiation (GO:0048712)	GO_Biological_Process_2015
Positive regulation of astrocyte activation (GO:0061890)	GO_Biological_Process_2017b
Positive regulation of astrocyte chemotaxis (GO:2000464)	GO_Biological_Process_2017b
Positive regulation of astrocyte differentiation (GO:0048711)	GO_Biological_Process_2023
Positive regulation of astrocyte differentiation (GO:0048711)	GO_Biological_Process_2017
Positive regulation of astrocyte differentiation (GO:0048711)	GO_Biological_Process_2017b
Positive regulation of astrocyte differentiation (GO:0048711)	GO_Biological_Process_2018
Positive regulation of astrocyte differentiation (GO:0048711)	GO_Biological_Process_2021
Positive regulation of astrocyte differentiation (GO:0048711)	GO_Biological_Process_2015
Regulation of astrocyte activation (GO:0061888)	GO_Biological_Process_2021
Regulation of astrocyte chemotaxis (GO:2000458)	GO_Biological_Process_2017b
Regulation of astrocyte differentiation (GO:0048710)	GO_Biological_Process_2023
Regulation of astrocyte differentiation (GO:0048710)	GO_Biological_Process_2018
Regulation of astrocyte differentiation (GO:0048710)	GO_Biological_Process_2021
Regulation of astrocyte differentiation (GO:0048710)	GO_Biological_Process_2015
Microglia differentiation (GO:0014004)	GO_Biological_Process_2017b
Microglial cell activation (GO:0001774)	GO_Biological_Process_2017
Microglial cell activation (GO:0001774)	GO_Biological_Process_2017b
Microglial cell activation (GO:0001774)	GO_Biological_Process_2018
Microglial cell activation (GO:0001774)	GO_Biological_Process_2021
Microglial cell activation (GO:0001774)	GO_Biological_Process_2023
Microglial cell activation (GO:0001774)	GO_Biological_Process_2015
Microglial cell activation involved in immune response (GO:0002282)	GO_Biological_Process_2017b
Negative regulation of microglial cell activation (GO:1903979)	GO_Biological_Process_2021
Negative regulation of microglial cell activation (GO:1903979)	GO_Biological_Process_2023
Positive regulation of microglia differentiation (GO:0014008)	GO_Biological_Process_2017b
Positive regulation of microglial cell activation (GO:1903980)	GO_Biological_Process_2017b
Positive regulation of microglial cell activation (GO:1903980)	GO_Biological_Process_2018
Positive regulation of microglial cell activation (GO:1903980)	GO_Biological_Process_2021
Positive regulation of microglial cell activation (GO:1903980)	GO_Biological_Process_2023
Positive regulation of microglial cell migration (GO:1904141)	GO_Biological_Process_2021
Positive regulation of microglial cell migration (GO:1904141)	GO_Biological_Process_2023
Regulation of microglial cell activation (GO:1903978)	GO_Biological_Process_2018
Regulation of microglial cell activation (GO:1903978)	GO_Biological_Process_2021
Regulation of microglial cell activation (GO:1903978)	GO_Biological_Process_2023
Regulation of microglial cell mediated cytotoxicity (GO:1904149)	GO_Biological_Process_2021
Regulation of microglial cell migration (GO:1904139)	GO_Biological_Process_2021
Regulation of microglial cell migration (GO:1904139)	GO_Biological_Process_2023

Supplementary Figures

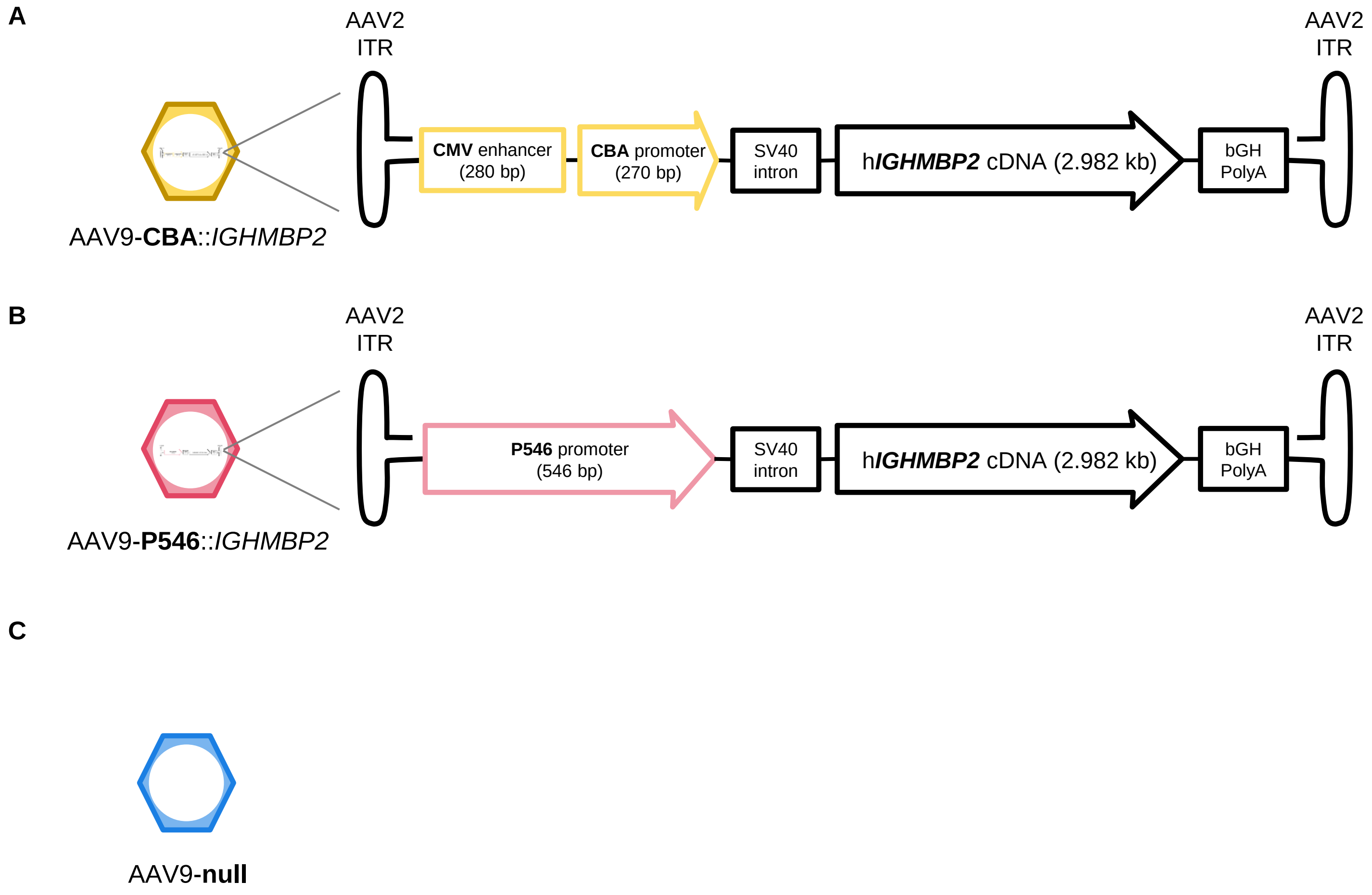

Figure S1. Schematic representation of tested AAV9-packaged constructs design
AAV9-CBA::*IGHMBP2* (A), AAV9-P546::*IGHMBP2* (B), and AAV9-null (C) constructs were shown. The therapeutic constructs (A-B) contain the human *IGHMBP2* cDNA under the control of CBA or P546 promoter, while AAV9-null is an empty AAV9 capsid, and considered as negative control (C). Abbreviations: adeno-associated virus serotype 9 (AAV9), chicken β-actin (CBA), cytomegalovirus (CMV), Inverted Terminal Repeats (ITR) of adeno-associated virus serotype 2 (AAV2), modified simian virus 40 (SV40) intron, bovine growth hormone (bGH), *Immunoglobulin mu DNA binding protein 2* (*IGHMBP2*).

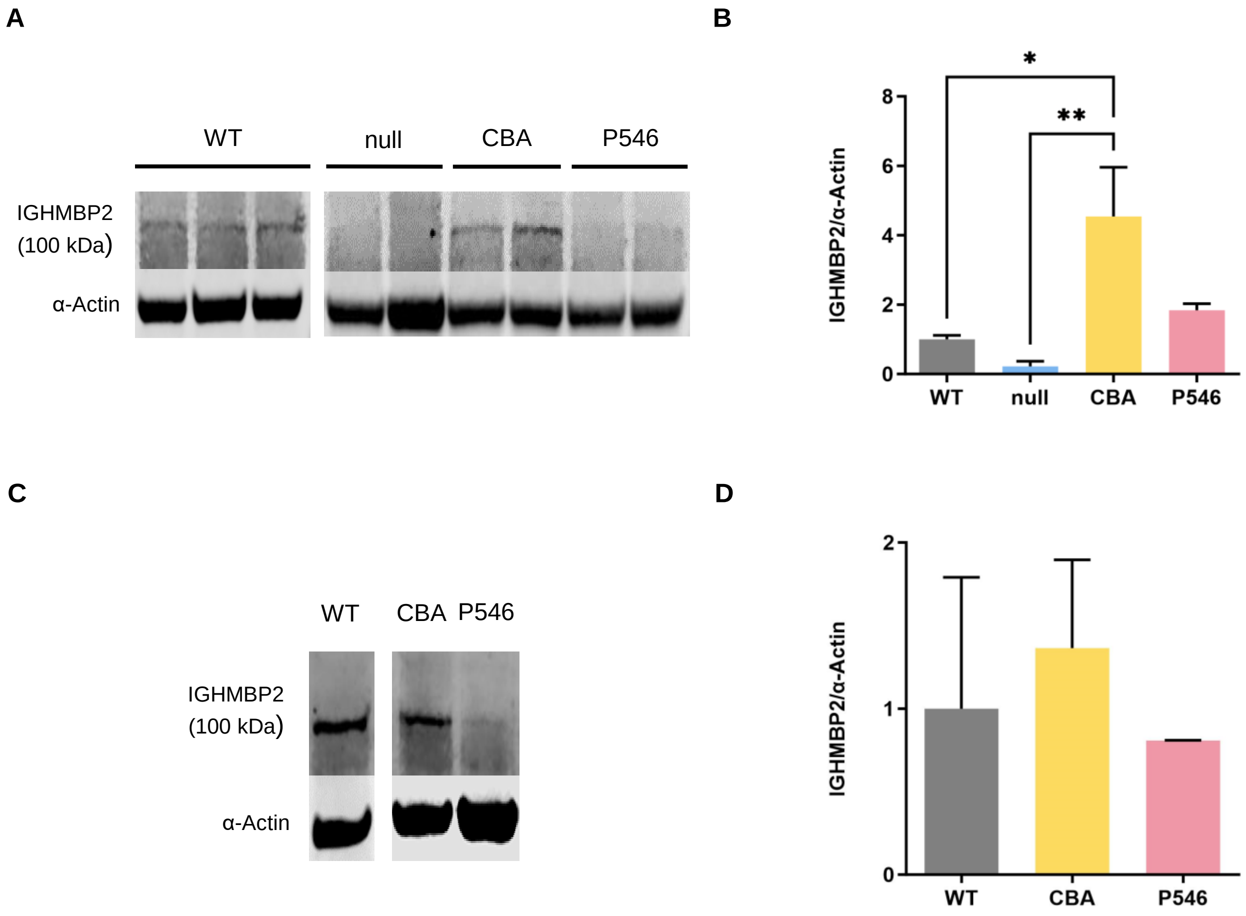

Figure S2. IGHMBP2 protein expression in mice spinal cord.
Representative WB analysis for IGHMBP2 protein performed on P20 (A) or P200 (C) spinal cords of WT mice and *nmd* mice treated ICV at P1 with the three constructs. (B-D) Relative densitometric evaluation normalized on α-Actin (n=4 animals/group). The values are represented as mean value ± SEM. Statistical analysis was performed with one-way ANOVA followed by Tukey's post hoc test. **P<0.01, *P<0.05

A

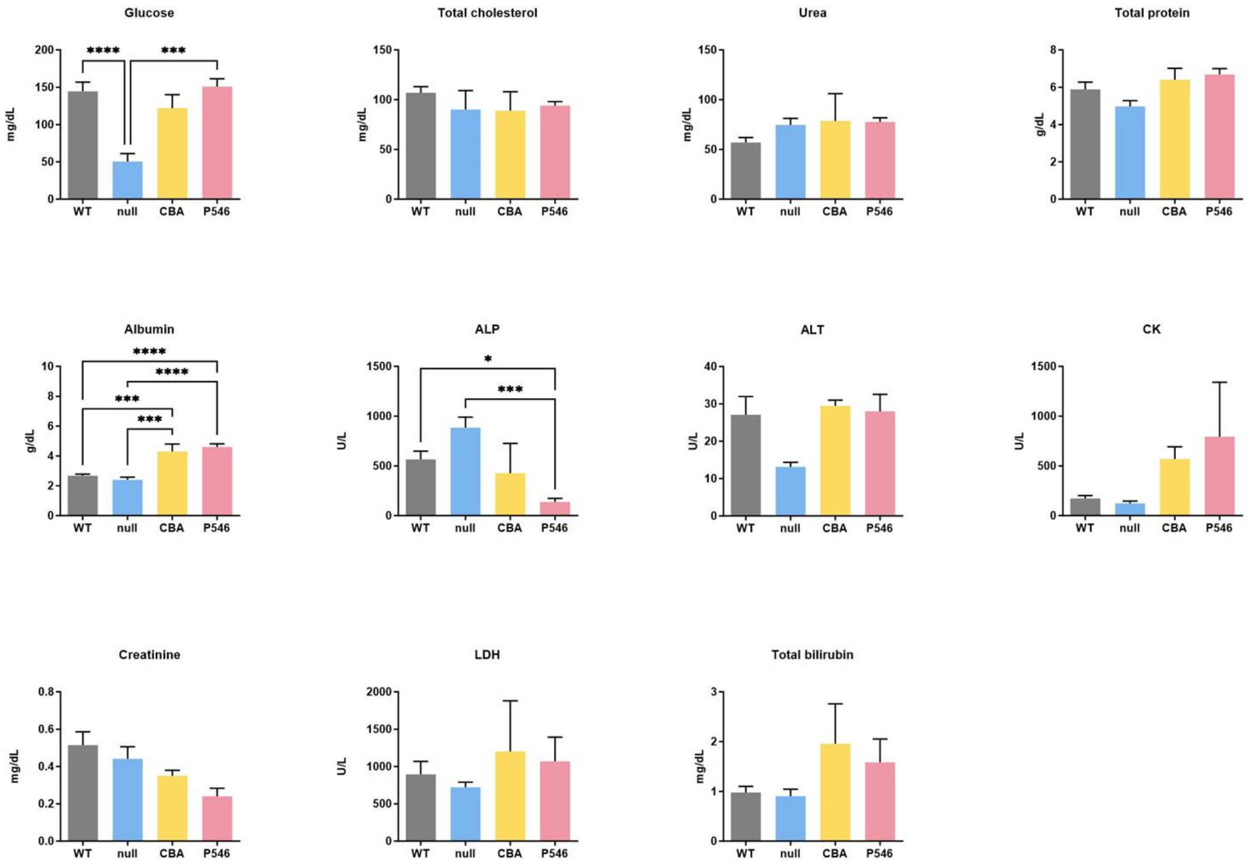

Figure S3. IGHMBP2 serum toxicity analysis.

(A) Serum concentration of toxicity biomarkers in WT, and nmd treated animals at P20 (n=4/group). Values are presented as means ± SEM. Statistical significance was determined by one-way ANOVA followed by Tukey's post hoc test. ****$P<0.0001$, ***$P<0.001$, *$P<0.05$

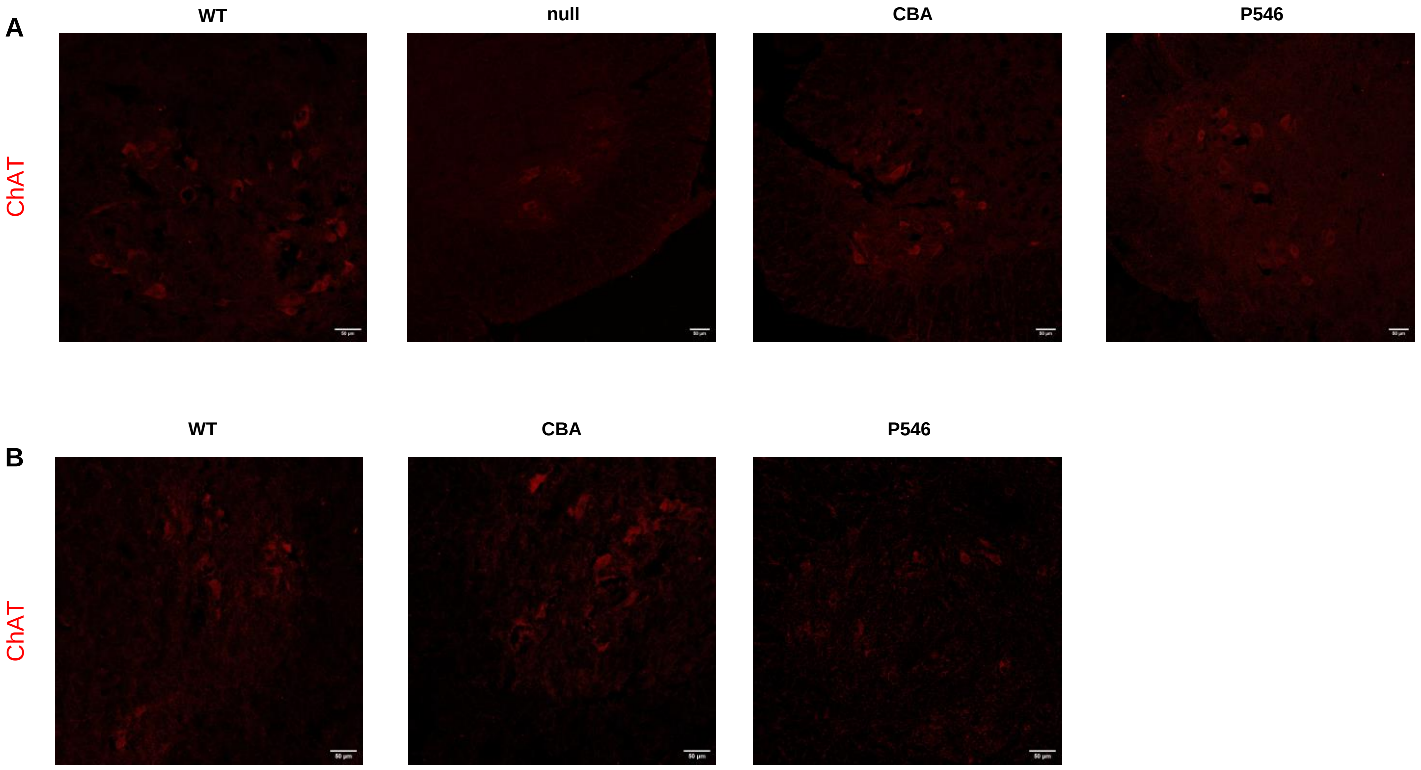

Figure S4. Chat positive MN distribution in mice spinal cord.
(A) Representative immunofluorescence images of 20 µm spinal cord's ventral horn sections from P20 WT and nmd treated mice stained for ChAT (red). Scale bar = 50 µm. (B) Representative immunofluorescence images of 20 µm spinal cord's ventral horn sections from adult WT and nmd treated mice stained for ChAT (red). Scale bar = 50 µm.

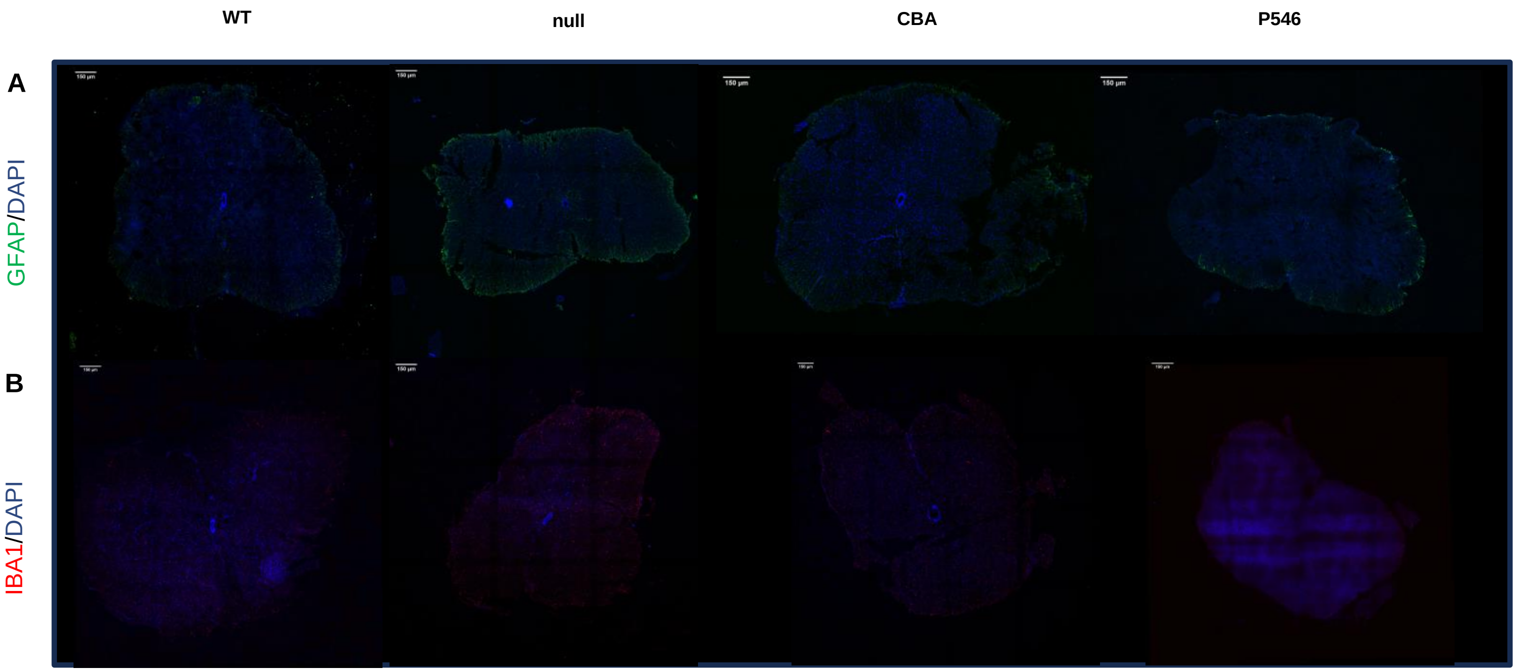

Figure S5. Astrocyte gliosis and microglial activation distribution in mice spinal cord. (A,B) Representative immunofluorescence images of 20 μm spinal cord sections of P20 WT and nmd treated mice stained for GFAP (green) (A) or IBA1 (red) (B) and DAPI (blue). TILE scanning.

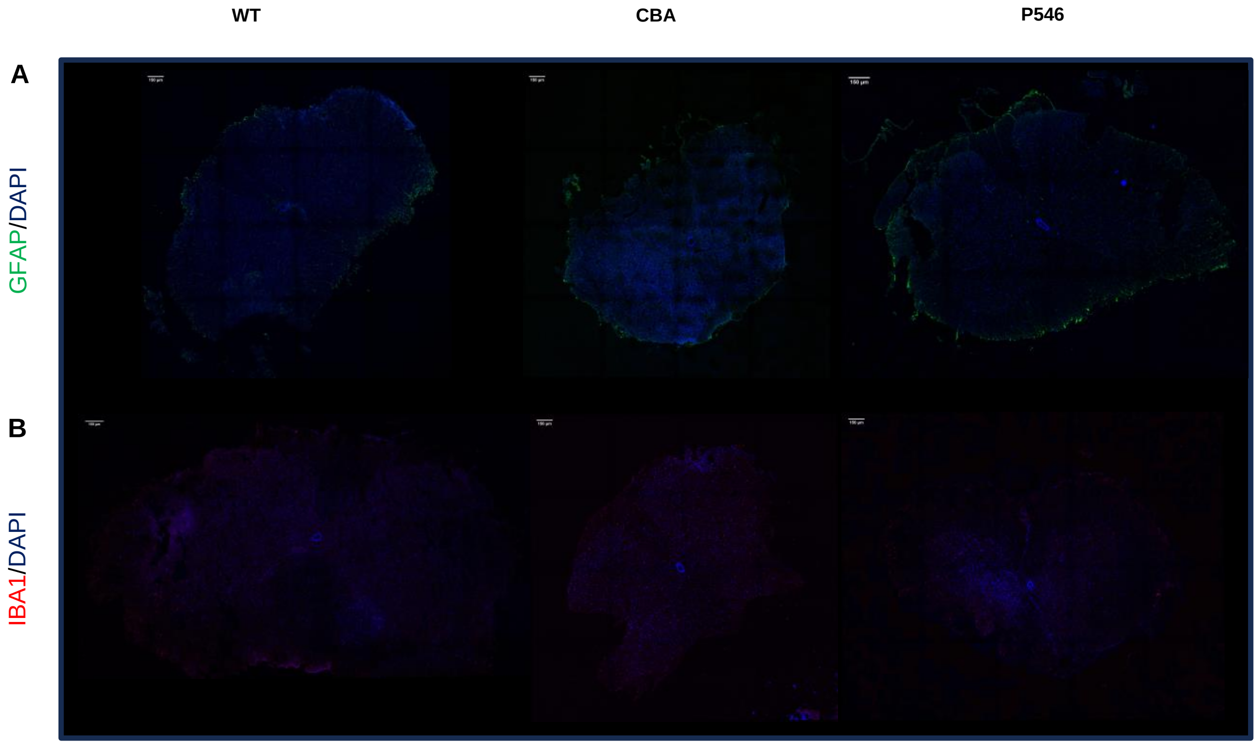

Figure S6. Astrocyte gliosis and microglial activation distribution in mice spinal cord. (A,B) Representative immunofluorescence images of 20 µm spinal cord sections of adult WT and nmd treated mice stained for GFAP (green) (A) or IBA1 (red) (B) and DAPI (blue). TILE scanning.

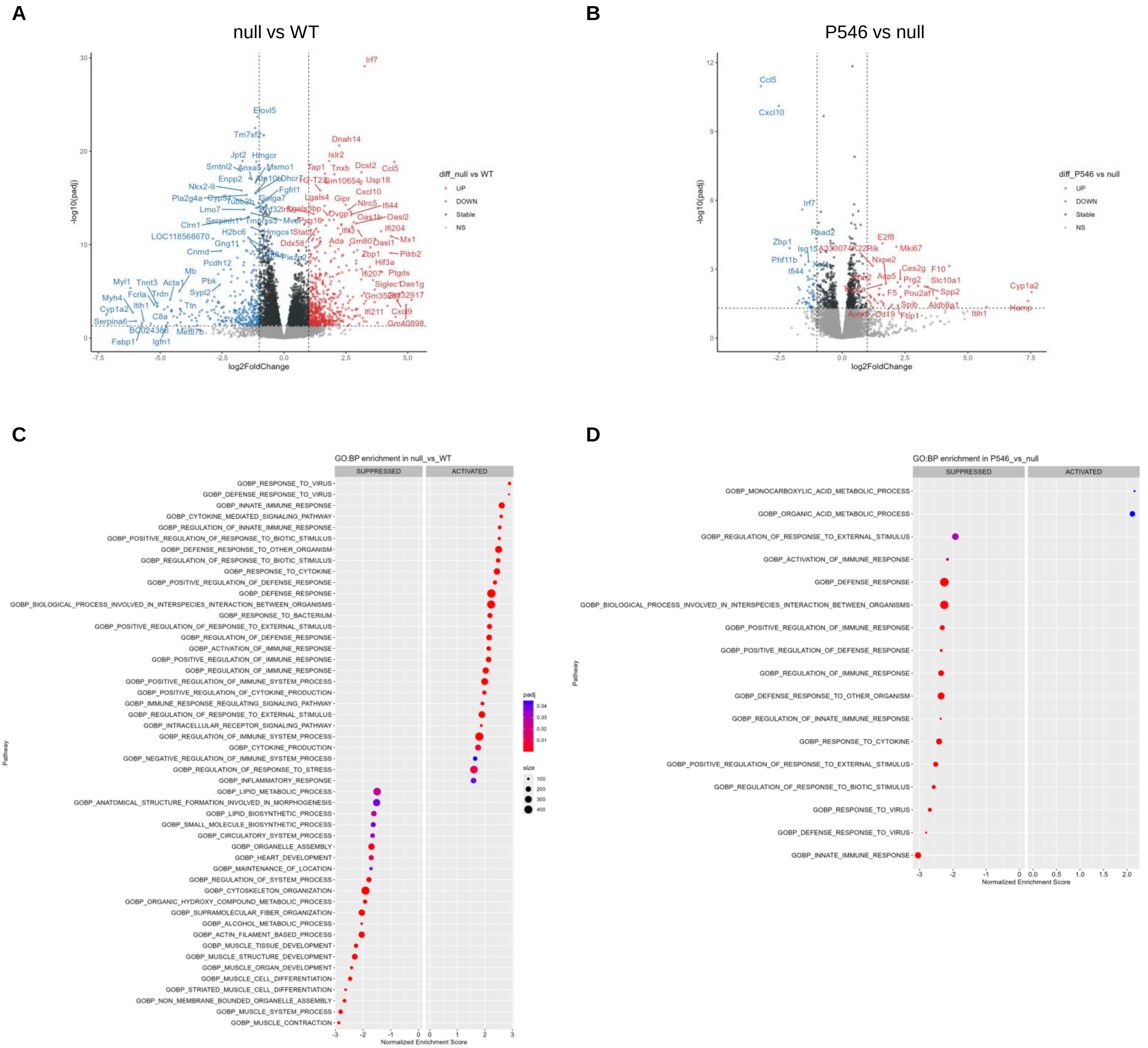

Figure S7: RNAseq GO enrichment.

(A-B) Volcano Plot of differentially expressed genes between null and WT (A) and P546 and null (B). GO enrichment analysis for upregulated and downregulated genes between WT vs *nmd* mice receiving null vector (C) and P546-treated animals vs null-treated (D). The gene designations and their ontology are indicated at the right of the heatmap.

Supplementary Materials

Video S1. Phenotypic appearance of gene therapy treated *nmd* mice.
Representative video of mice treated with either CBA or P546 vector ICV at P1 examined at P100 with their WT littermates.

References:

44. Bersani, M., Rizzuti, M., Pagliari, E., Garbellini, M., Saccomanno, D., Moulton, H.M., Bresolin, N., Comi, G.P., Corti, S., and Nizzardo, M. (2022). Cell-penetrating peptide-conjugated Morpholino rescues SMA in a symptomatic preclinical model. Mol. Ther. *30*, 1288–1299. https://doi.org/10.1016/j.ymthe.2021.11.012.

49. Moderated estimation of fold change and dispersion for RNA-seq data with DESeq2 | Genome Biology | Full Text https://genomebiology.biomedcentral.com/articles/10.1186/s13059-014-0550-8.
